# Primary yolk sac tumor of pterygopalatine fossa with loss of vision: A case report

**DOI:** 10.1097/MD.0000000000024916

**Published:** 2021-02-26

**Authors:** Ye-Hua Shen, Shou-Yin Jiang

**Affiliations:** a Department of Radiology, The Children's Hospital, Zhejiang University School of Medicine, National Clinical Research Center for Child Heath; b Department of Emergency Medicine, The Second Affiliated Hospital, Zhejiang University School of Medicine; Research Institute of Emergency Medicine, Zhejiang University, Hangzhou, China.

**Keywords:** alpha-fetoprotein, alpha-fetoprotein, extragonadal germ cell tumors, imaging, loss of vision, yolk sac tumor

## Abstract

**Introduction:**

Primary yolk sac tumor (YST) is an infrequently-diagnosed malignant extragonadal germ cell tumors. It is likely to recur locally and may present with widespread metastases once diagnosed. Primary YST of the head is uncommon but can cause severe complications, such as loss of vision once the tumor mass invades the optic nerve.

**Patient concerns:**

A 20-month-old boy presented to the general clinic of the local children's hospital with a complaint of swelling of left face for 1 year and proptosis of the left eye for over 2 weeks as stated by his parents. Initially, he did have some vision, as he could walk by himself, but a special ophthalmologic examination was not performed.

**Diagnoses:**

Cranial computed tomography and magnetic resonance imaging revealed a large tumor accompanied by peripheral bone destruction in the left pterygopalatine fossa that extended to sphenoid, ethmoid, left maxillary sinuses, left nasoethmoid, and left orbit. The optic nerve was invaded on both sides. Chest and abdominal imaging were normal. A primary diagnosis of Langerhans cell hyperplasia was made. However, blood tests on the second day of hospitalization revealed significantly elevated serum alpha-fetoprotein levels. On the third day, the boy lost his eyesight, with loss of pupillary and no light sensation during flashlight stimulation on both sides.

**Interventions:**

Nasal endoscopy was performed on the fourth day, the vast majority of soft tissue mass was resected for biopsy. Histopathological examination revealed features of endodermal sinus tumor. A final diagnosis of primary YST of pterygopalatine fossa was made. Because the mass could not be resected completely, he received combined chemotherapy with bleomycin, etoposide, and carboplatin for 6 cycles over six months.

**Outcomes:**

The patient recovered with significant tumor shrinkage and without secondary metastasis after 18 months but left permanently blind.

**Conclusion:**

The worst complication of loss of vision after Primary YST of pterygopalatine fossa alerts us that close physical examination during the initial investigation should be performed, which is especially important in young children who cannot express complaints well. Early detection and treatment with surgical resection and chemotherapy may contribute to satisfactory outcomes and avoidance of visual impairment.

## 1 Introduction

Primary yolk sac tumor (YST) is an infrequent malignant germ cell tumor that mainly originates from the gonads and exhibits a high degree of tissue invasiveness. Alpha-fetoprotein (AFP), imaging methods, and histopathological examination are effective diagnostic and monitoring approaches for the comprehensive management of YST. The incidence of primary YST of head and neck is only 5%.^[1]^ Although uncommon and not previously reported, primary YST of pterygopalatine fossa can cause severe complications, such as loss of vision, once the tumor mass invades the optic nerve. In this report, we present a case of YST of pterygopalatine fossa in a child who ultimately developed blindness, although the tumor was satisfactorily treated with combined surgery and chemotherapy. Physicians and parents should learn a lesson from this case.

## 2 Case presentation

A 20-month-old boy was referred to the general clinic of the local children's hospital with a complaint of swelling of left face for 1 year and proptosis of the left eye for over two weeks as stated by his parents. Initially, he did have some vision because he was able walk by himself without falling. The patient did not complain of any other symptoms. His symptoms started 1 year ago when his left face exhibited mild swelling that was noticed by his parents. The swelling had worsened recently, and perceived proptosis of the left eye was noted two weeks prior. The patient had unremarkable previous medical history.

On examination, he was conscious with evident proptosis of the left eye, and light swelling of the left face was noted without tenderness. Eye movement on both sides was normal. Unfortunately, initial pupillary reflex examinations were not performed, and the vision condition was not assessed. Other physical examinations were globally normal, including normal myodynamia and superficial and deep sensation as well as negative Babinski sign.

**Figure 1:**
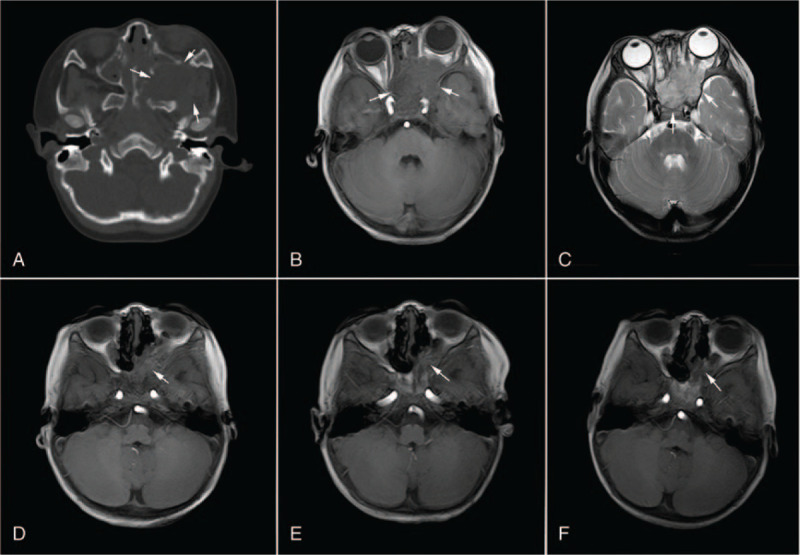
Dynamic changes of yolk sac tumor of pterygopalatine fossa on computerized tomography and magnetic resonance imaging before and after surgery in combination with chemotherapy. A: Tumor mass is accompanied by peripheral bone destruction around the left pterygopalatine fossa before surgery (white arrow); B and C: Yolk sac tumor of the left pterygopalatine fossa with obstructive changes in the sphenoid, ethmoid, left maxillary sinuses, left nasal cavity, and left orbit (white arrow), and the bilateral optic nerve was invaded; D: Residual tumor mass after massive resection and 3 cycles of chemotherapy; E, Tumor mass at the end of 6 cycles of chemotherapy; F, Tumor mass 18 months after index admission.

Cranial computerized tomography (CT) showed a large tumor accompanied by peripheral bone destruction around the left pterygopalatine fossa (Fig. 1A). Brain magnetic resonance imaging (MRI) revealed a left pterygopalatine fossa mass with obstructive changes in the sphenoid, ethmoid, left maxillary sinuses, left nasal cavity, and left orbit. The mass exhibited relatively heterogeneous signal intensities and intense enhancement on the contrast MRI (Fig. 1B, C). The optic nerve was invaded on both sides. A chest CT and abdominal ultrasonography did not reveal any abnormal findings. Based on age and imaging findings, a primary diagnosis of Langerhans cell hyperplasia was made. However, a definite diagnosis should be based on histopathology. He was hospitalized in the Oncological Surgery department for further operation preparation.

Blood routine, C-reactive protein, blood biochemistries, liver function, and urine analyses were normal. On the second day of hospitalization, blood tests revealed that the serum AFP level was markedly elevated (10617 ng/mL, reference range: 0–20 ng/mL).

**Figure 2:**
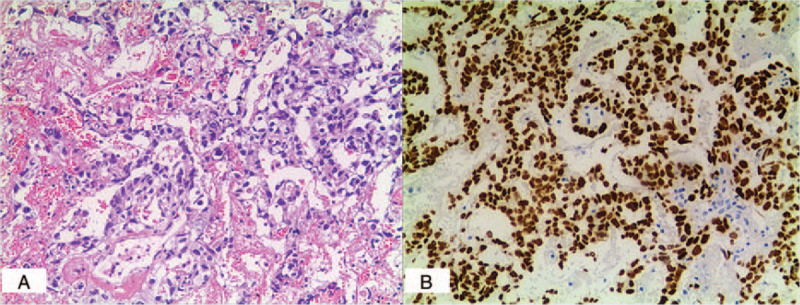
A: Tumor mass of the middle nasal meatus (×100). Microscopically, tumor components were scattered among the fibrous tissue, showing microcapsule-like or papillary arrangement, obvious cell atypia, nuclear fission, regional bleeding and necrosis. B: Immunohistochemical staining for sal-like protein 4 (×100).

On the third day of hospitalization before surgery, the boy lost his eyesight, and eye examination revealed loss of pupillary reflex in both eyes with a bilateral pupil diameter of 4.0 mm. No light sensation was observed during flashlight stimulation on both sides. On the fourth day, the patient underwent exploration, resection and biopsy of the soft tissue mass through nasal endoscopy. The resected specimen appeared as a dark red neoplasm that was brittle and easily bled. Due to the deep location, the mass could not be resected completely. Biopsy confirmed morphologic and immunohistochemical findings of YST, and immunohistochemical stain was diffusely positive for sal-like protein 4, AFP, placental alkaline phosphatase, creatine kinase, and vimentin (Fig. 2). The final diagnosis of the presented case was primary YST. On day 3 following surgery, the patient received a total of 6 cycles of neo-adjuvant chemotherapy consisting of bleomycin (6000 units for one day), etoposide (32 mg daily for 4 days), and carboplatin (80 mg daily for 3 days) for six months. No significant adverse effects were noted during chemotherapy. After the completion of the third cycle, the serum AFP level was completely normalized. MRI of the ill-defined area exhibited a gross residual lesion (Fig. 1D). After treatment, MRI showed significant reduction of the tumor mass (Fig. 1E, F), and AFP levels were normal for the remaining days. Chest CT scanning and abdominal ultrasonography did not identify any metastasis 18 months after index admission. Collectively, the patient recovered with significant tumor shrinkage and without secondary metastasis. However, his eyesight was not restored.

This study was approved by the ethics committee of the Second Affiliated Hospital of Zhejiang University School of Medicine. Written informed consent was obtained from the patient for the publication of this case report and accompanying images.

## 3 Discussion

YST is the leading histologic type among malignant extragonadal germ cell tumors (EGCTs), which occur most commonly in association with teratoma and rarely develop separately. YST occurs much more frequently in female children. Previous studies have reported YST of the left pterygoid region,^[1]^ upper lip,^[2]^ parapharyngeal and skull base,^[3]^ posterior fossa,^[4]^ floor of the mouth,^[5]^ and right masticator space.^[6]^ To the authors’ knowledge, there has been no previous report of primary YST of pterygopalatine fossa.

The pathogenesis remains unknown. However, two hypotheses have been proposed regarding the origin of EGCTs. First, EGCTs, which develop from primordial germ cells, are generated as a result of defects in the cell migration process during embryonic development. Primordial germ cells in the 4-week-old embryo present at first in the wall of the yolk sac and migrate through the dorsal mesentery to the genital ridge during embryogenesis.^[7]^ However, some germ cells will not complete this migration, which results in retention at certain sites near the midline. Second, EGCTs may develop from totipotent cells, which are distributed dispersedly in the entire body during embryonic development and remain dormant under normal conditions. When stimulated, these cells may differentiate to EGCTs.

YST can be underdiagnosed when imaging examination is exclusively used, but integrating histological and immunohistochemical examinations can improve the diagnosis. CT and MRI images are useful for identifying the location and invasion range of the mass. According to our experience, a soft tissue mass along with bone destruction indicates that it may be a common disease in children, and we initially had a presumptive diagnosis of Langerhans histiocytosis. However, the tumor marker serum AFP was significantly increased. AFP is a glycoprotein that is produced primarily by yolk sac and liver during fetal development. Thus, we suspected that the lesion must be a reproductive tumor. EGCTs secrete basic fetal markers such as AFP and are highly chemo-sensitive. Thus, serum AFP is also a useful tumor biomarker for assessment for the therapeutic effect and predictions of recurrence, residue, and metastasis during the follow-up period in patients with YST. When suspected, the diagnosis can be easily confirmed by histopathology with immunohistochemistry. The major pathologic characteristics of YSTs include Schiller-Duval bodies, a festoon-like pattern, a hepatoid pattern with hyaline globules, and a polyvesicular vitelline pattern^[2]^. Immunohistochemically identified AFP is a pivotal biomarker for the diagnosis of YST.

YST generally exhibits poor prognosis due to its tendency for local recurrence and high degree of invasiveness and metastasis. Given that the tumor mass can be absolutely unresectable at diagnosis, multimodal therapy combining surgery, chemotherapy, and radiotherapy have been used in the past.^[8]^ However, the prognosis of EGCTs in the head and neck region is uncertain. Thus, Filho et al. advocated surgical resection with radiotherapy for nasal EGCTs, yielding a satisfactory outcome in a 48-year-old man.^[9]^ The patient showed no evidence of metastasis 7 years after treatment. However, not all patients exhibit optimal results. A 3-year-old girl with an EGCT in the orbit/maxillary received chemotherapy and resection of the mass, but the tumor recurred massively 8 weeks following treatment.^[10]^ Gabris et al. demonstrated that radiotherapy might have led to the maldevelopment of teeth.^[10]^ A multidisciplinary approach to treatment including surgical resection and adjuvant chemotherapy has been recommended for most EGCTs of the head and neck region. A multiagent chemotherapeutic cocktail regimen, including bleomycin, etoposide, cisplatin/carboplatin, cyclophosphamide, ifosfamide, vincristine and 5-fluorouracil, has significantly improved the outcome of these patients. In our patient, aggressive surgical excision followed by chemotherapy provided good outcome based on the recent visit.

In the current case, the patient developed blindness shortly after hospitalization, which constitutes a severe complication of YST of pterygopalatine fossa that has not been reported previously. Delay in seeking medical care in this circumstance facilitates the growth and invasion of the tumor mass, which should alert parents when a child develops visual impairment and proptosis. In young children, the optimal window for diagnosis may be missed due to the inability to express complaints; thus, physicians should perform a careful and comprehensive physical examination. Nothing is more important than early detection and early treatment in preventing the tragedy of blindness.

In summary, primary YST of pterygopalatine fossa occurs rarely but may have poor prognosis due to its high degree of invasiveness and metastasis and the difficulties encountered in obtaining complete resection. The worst complication of loss of vision alerts us of the importance of performing a close physical examination during the initial investigation, which is especially important in small children who cannot express complaints. Early detection of YST of pterygopalatine fossa and treatment with surgical resection and adjuvant chemotherapy may contribute to satisfactory outcomes (tumor shrinkage and restricting tumor metastasis) and avoidance of visual impairment.

## Author contributions

**Conceptualization**: Shou-Yin Jiang

**Investigation**: Ye-Hua Shen, Shou-Yin Jiang

**Methodology**: Ye-Hua Shen, Shou-Yin Jiang

**Supervision**: Ye-Hua Shen, Shou-Yin Jiang

**Validation**: Ye-Hua Shen

**Visualization**: Ye-Hua Shen, Shou-Yin Jiang

**Writing – original draft**: Ye-Hua Shen

**Writing – review & editing**: Shou-Yin Jiang
